# Supplementary material for: A Critical View of the Application of the APEX Software (Aqueous Photochemistry of Environmentally-Occurring Xenobiotics) to Predict Photoreaction Kinetics in Surface Freshwaters
Source: Molecules. 2019 Dec 18;25(1):9. doi: 10.3390/molecules25010009 (PMC7017383; doi:10.3390/molecules25010009)
Supplement: Supplementary file 1 [file molecules-25-00009-s001.zip › 3.2.4_gcc-4.4.0.zip!/3.2.4_gcc-4.4.0/3.2.4_gcc-4.4.0/doc/octave/html/interpreter/Creating-Cell-Arrays.html]

Creating Cell Arrays - Untitled


Next: Indexing Cell Arrays,
Previous: Basic Usage of Cell Arrays,
Up: Cell Arrays


---

#### 6.2.2 Creating Cell Array

The introductory example (see Basic Usage of Cell Arrays) showed
how to create a cell array containing currently available variables.
In many situations, however, it is useful to create a cell array and
then fill it with data.

The `cell` function returns a cell array of a given size, containing
empty matrices. This function is similar to the `zeros`
function for creating new numerical arrays. The following example creates
a 2-by-2 cell array containing empty matrices

```
     c = cell(2,2)
          => c =
     
              {
                [1,1] = [](0x0)
                [2,1] = [](0x0)
                [1,2] = [](0x0)
                [2,2] = [](0x0)
              }
```

Just like numerical arrays, cell arrays can be multidimensional. The
`cell` function accepts any number of positive integers to describe
the size of the returned cell array. It is also possible to set the size
of the cell array through a vector of positive integers. In the
following example two cell arrays of equal size are created, and the size
of the first one is displayed

```
     c1 = cell(3, 4, 5);
     c2 = cell( [3, 4, 5] );
     size(c1)
          => ans =
              3   4   5
```

As can be seen, the `size` function also works
for cell arrays. As do other functions describing the size of an
object, such as `length`, `numel`, `rows`, and `columns`.

— Built-in Function: **cell** (x)  
— Built-in Function: **cell** (n, m)  
> Create a new cell array object. If invoked with a single scalar
> argument, `cell` returns a square cell array with the dimension
> specified. If you supply two scalar arguments, `cell` takes
> them to be the number of rows and columns. If given a vector with two
> elements, `cell` uses the values of the elements as the number of
> rows and columns, respectively.

As an alternative to creating empty cell arrays, and then filling them, it
is possible to convert numerical arrays into cell arrays using the
`num2cell` and `mat2cell` functions.

— Loadable Function: c = **num2cell** (m)  
— Loadable Function: c = **num2cell** (m, dim)  
> Convert the matrix m to a cell array. If dim is defined, the
> value c is of dimension 1 in this dimension and the elements of
> m are placed in slices in c.
>
>
>
> **See also:** mat2cell.

— Loadable Function: b = **mat2cell** (a, m, n)  
— Loadable Function: b = **mat2cell** (a, d1, d2, ...)  
— Loadable Function: b = **mat2cell** (a, r)  
> Convert the matrix a to a cell array. If a is 2-D, then
> it is required that `sum (`m`) == size (`a`, 1)` and
> `sum (`n`) == size (`a`, 2)`. Similarly, if a is
> a multi-dimensional and the number of dimensional arguments is equal
> to the dimensions of a, then it is required that `sum (`di`)
> == size (`a`, i)`.
>
> Given a single dimensional argument r, the other dimensional
> arguments are assumed to equal `size (`a`,`i`)`.
>
> An example of the use of mat2cell is
>
> ```
>           mat2cell (reshape(1:16,4,4),[3,1],[3,1])
>           => {
>             [1,1] =
>           
>                1   5   9
>                2   6  10
>                3   7  11
>           
>             [2,1] =
>           
>                4   8  12
>           
>             [1,2] =
>           
>               13
>               14
>               15
>           
>             [2,2] = 16
>           }
> ```
>
>
>
>
> **See also:** num2cell, cell2mat.
